# Preferences for attributes of an artificial intelligence-based risk assessment tool for HIV and sexually transmitted infections: a discrete choice experiment

**DOI:** 10.1186/s12889-024-20688-2

**Published:** 2024-11-21

**Authors:** Phyu M. Latt, Nyi N. Soe, Alicia J. King, David Lee, Tiffany R. Phillips, Xianglong Xu, Eric P. F. Chow, Christopher K. Fairley, Lei Zhang, Jason J. Ong

**Affiliations:** 1 Artificial Intelligence and Modelling in Epidemiology Program, Melbourne Sexual Health Centre, Alfred Health, Melbourne, Australia; 2 School of Translational Medicine, Faculty of Medicine, Nursing and Health Sciences, Monash University, Melbourne, Australia; 3 Melbourne Sexual Health Centre, Alfred Health, Melbourne, Australia; 4 School of Public Health, Shanghai University of Traditional Chinese Medicine, Shanghai, China; 5 Centre for Epidemiology and Biostatistics, Melbourne School of Population and Global Health, The University of Melbourne, Melbourne, Australia; 6 Clinical Medical Research Center, Children’s Hospital of Nanjing Medical University, Nanjing, Jiangsu Province, China; 7 Department of Clinical Research, London School of Hygiene and Tropical Medicine, London, UK

## Abstract

**Introduction:**

Early detection and treatment of HIV and sexually transmitted infections (STIs) are crucial for effective control. We previously developed *MySTIRisk*, an artificial intelligence-based risk tool that predicts the risk of HIV and STIs. We examined the attributes that encourage potential users to use it.

**Methods:**

Between January and March 2024, we sent text message invitations to the Melbourne Sexual Health Centre (MSHC) attendees to participate in an online survey. We also advertised the survey on social media, the clinic's website, and posters in affiliated general practice clinics. This anonymous survey used a discrete choice experiment (DCE) to examine which *MySTIRisk* attributes would encourage potential users. We analysed the data using random parameters logit (RPL) and latent class analysis (LCA) models.

**Results:**

The median age of 415 participants was 31 years (interquartile range, 26–38 years), with a minority of participants identifying as straight or heterosexual (31.8%, *n* = 132). The choice to use *MySTIRisk* was most influenced by two attributes: cost and accuracy, followed by the availability of a pathology request form, level of anonymity, speed of receiving results, and whether the tool was a web or mobile application. LCA revealed two classes: "The Precisionists" (66.0% of respondents), who demanded high accuracy and "The Economists" (34.0% of respondents), who prioritised low cost. Simulations predicted a high uptake (97.7%) for a tool designed with the most preferred attribute levels, contrasting with lower uptake (22.3%) for the least preferred design.

**Conclusions:**

Participants were more likely to use *MySTIRisk* if it was free, highly accurate, and could send pathology request forms. Tailoring the tool to distinct user segments could enhance its uptake and effectiveness in promoting early detection and prevention of HIV and STIs.

**Supplementary Information:**

The online version contains supplementary material available at 10.1186/s12889-024-20688-2.

## Introduction

Sexually transmitted infections (STIs) pose a significant global public health concern, with more than one million new cases reported every day in 2020 and an estimated 374 million yearly cases of curable infections worldwide [1]. These numbers from the World Health Organization (WHO) highlight the need for action to mitigate the public health burden of STIs. Reflecting this global concern, Australia has experienced a noticeable rise in STI rates over the past decade. Specifically, Australia has witnessed a tripling in syphilis diagnoses, a doubling of gonorrhoea diagnoses, and a 12% increase in chlamydia infections since 2013 [2].

One of the major challenges in the treatment and control of STIs lies mostly in their asymptomatic nature, where infected individuals may not exhibit noticeable symptoms. The WHO estimates that most STIs are asymptomatic, leading to unawareness among infected individuals and inadvertent transmission [1]. If left untreated, asymptomatic STIs can lead to severe complications, such as infertility, and are associated with an increased HIV risk [3]. Therefore, early detection and treatment are crucial to prevent harm and further spread. This underscores the need for effective screening tools that enable individuals to assess their risk of HIV/STIs so that they can receive early appropriate testing.

To address this need, the Melbourne Sexual Health Clinic (MSHC) has developed *MySTIRisk*, using artificial intelligence (AI) to provide individualised risk assessment for three common STIs: syphilis, chlamydia and gonorrhoea and HIV [4–6]. The individualised risk scores generated by *MySTIRisk* enable the more efficient identification of high-risk individuals, facilitating targeted screening to identify asymptomatic infections that might otherwise be missed. Exploratory qualitative research with users suggests the accessibility and acceptability of this tool are likely to be impacted by the complex interplay of various attributes, such as cost, speed, accuracy, and anonymity [7]. However, there is limited evidence on what users value most in AI-based HIV/STI risk assessment tools.

A number of study designs have been adopted to understand the preferences of the users of health interventions. The discrete choice experiment is a WHO-endorsed methodology [8] for eliciting preferences for health interventions. Integrating DCEs into research allows exploration of how individuals assign value to specific attributes of services or goods [9, 10]. DCEs have become widely adopted in health economics for eliciting preferences, especially regarding new products or services not yet available on the market [11]. Moreover, DCEs quantitatively measure preferences, offering nuanced insights at subpopulation levels.

Our study aimed to identify the key features that influence the future uptake of the *MySTIRisk* tool, utilising a DCE, and examine how individuals trade-off between these attributes. The study results will inform evidence-based adaptations of *MySTIRisk* to align with the needs and expectations of end-users, potentially leading to an increase in the tool's uptake and facilitating early testing and diagnosis.

## Methods

### Study population and recruitment

We used multiple methods to recruit individuals between January and March 2024 who were aged 18 years or older, living in Australia and reported having sex in the past 12 months. Eligible participants included all genders, sexes, and sexual orientations, including transgender and gender diverse (TGD) individuals. We sent a single short message service (SMS) invitation to each individual who had visited the Melbourne Sexual Health Centre (MSHC) and had previously consented to receive SMS communications. We identified eligible participants through daily queries of data collected using Computer-Assisted Self Interview (CASI), which was completed upon arrival at the MSHC. Eligible participants were then contacted via direct SMS messaging on the day after their visit. Additionally, we advertised the survey on social media platforms, including Facebook and X (formerly Twitter), the MSHC's website [12], and via posters in two general practice clinics that were affiliated with MSHC.

We used the Qualtrics online survey platform (Qualtrics, Provo, UT) to administer the survey. Participants were introduced to the study with an explanation of the risk assessment tool accompanied by relevant screenshots. Additionally, we outlined the purpose behind conducting this study. After reading the plain language statement, participants were asked to provide consent by selecting either the "Agree" or "Disagree" option. This step was necessary to proceed with the survey. If a participant clicked on "Disagree", the survey would end immediately. The complete questionnaire used in this study, including the discrete choice experiment, was developed specifically for this research and is available as Supplementary File 1.

The survey included questions on sociodemographic details such as age, gender, sexual identity and orientation, education, employment status, and comfort with technology (prior experience with the use of an online risk assessment tool). Additionally, it asked about past HIV/STI testing experiences and preferences for using an AI-powered risk assessment tool, explored through questions explicitly designed for this purpose (DCE). The survey was anonymous, and participants had the option to provide their telephone number and/or email in a separate survey to enter a draw for one of ten AU$50 vouchers.

### DCE Design

During the formative phase of the DCE, we conducted a literature review to identify potential attributes affecting the uptake of online tools [13–17], followed by qualitative interviews with 14 participants to identify motivations for using AI-based risk assessment tools for HIV and STIs [7]. Table S1 describes the final selected attributes and levels. We selected them based on their importance as identified in the literature and qualitative interviews, as well as their feasibility within the DCE framework. We excluded some potentially relevant attributes to maintain a focused and manageable DCE for participants. To reduce respondent fatigue, the experimental design comprised 12 choice sets divided into two blocks, allowing participants to answer six choice sets. Each choice set offered three unlabelled scenarios (A, B, and C), including an opt-out option for those preferring none of the choices (Table S2). We used NGENE Software (Version 1.4, Choicemetrics, USA) to construct a D-efficient experimental design to maximise the information from each set. A pilot survey was conducted with six participants (four male, two female), leading to minor adjustments in wording for enhanced clarity without altering the defined attributes or levels.

### Statistical analysis

We summarised participant sociodemographic characteristics using descriptive statistics in STATA (version 17, StataCorp). We analysed the choice data using the Random Parameters Logit (RPL) model in NLOGIT 6 (version 6, Econometric Software Inc, USA). We chose RPL considering the panel nature of our dataset (repeated observations per participant) and to relax the assumptions of a multinomial logit model [18, 19]. We used 1000 Halton draws, assuming normally distributed parameters. We assessed the model fit based on log-likelihood and Akaike information criteria.

We used effects coding for the attribute levels [20]. We used the simulation function within NLOGIT to estimate the probability of choosing the risk assessment tool under various scenarios (best case with preferred attributes, worst case, likely standard format) using choice data from the RPL models [21].

We conducted a latent class analysis (LCA) to identify preference clusters among participant subgroups. We used interaction terms to probe whether these clusters could be delineated based on a priori hypothesised observable characteristics (e.g., less than five years in Australia, higher education, and MSHC attendees). Regarding the LCA results, positive coefficients indicate a higher likelihood of choosing the tool when that attribute level is present compared to the reference level. The magnitude of the attribute coefficients reflects the relative importance of each attribute level's contribution to the overall utility. Statistical significance was assessed using a standard p-value threshold of 0.05.

## Results

### Study population

We sent 3153 text messages to eligible MSHC attendees between January and March 2024. Additionally, we recruited 59 participants through social media, including Facebook and X, the official MSHC website [12], and affiliated general practice clinics. Among those, 468 accessed the online survey. Of these, 53 were excluded from the analysis for the following reasons: 7 did not provide consent, and 46 reported no sexual activity in the past 12 months. The remaining 415 participants who completed the survey and met the eligibility criteria were included in the final analysis.[12] The median time to complete the DCE survey was 5 min (IQR: 3.5–7.1), and the participants chose the “opt-out” option (neither option A nor B) in 6.4% of 2490 choice sets.

The median age of the participants was 31 years (IQR 26–38 years). The majority self-identified as male (62.4%, *n* = 259), while 41.0% (*n* = 170) identified as lesbian, gay or homosexual. Participants had a high level of educational attainment, with most having a bachelor's degree or higher (68.0%, *n* = 282). Nearly half (47.0%, *n* = 195) were born in Australia. Most reported being very comfortable using mobile apps and websites (78.8%, *n* = 327) and had previously been tested for HIV/STIs (91.1%, *n* = 378). However, only a small proportion (14%, *n* = 58) had ever used an online risk assessment tool for STIs (see Table 1).

**Table 1:** Sociodemographic characteristics of the study population (*N* = 415)

	**Total (*N* = 415) (n, %)**	**Online Recruitment**^**a**^** (*N* = 59) (*n*, %)**	**Sexual health clinic (*N* = 356) (*n*, %)**
**Age, median (IQR)**	31 (26–38)	31 (26–38)	31 (26–37)
**Sex assigned at birth**			
Male	273 (65.78%)	25 (42.37%)	248 (69.66%)
Female	142 (34.22%)	34 (57.63%)	108 (30.34%)
**Gender**			
Male	259 (62.41%)	25 (42.37%)	234 (65.73%)
Female	130 (31.33%)	30 (50.85%)	100 (28.09%)
Non-binary/gender-fluid	22 (5.3%)	3 (5.08%)	19 (5.34%)
Another gender^b^	3 (0.72%)	1 (1.69%)	2 (0.56%)
Don’t know/Prefer not to answer	1 (0.24%)	0 (0%)	1 (0.28%)
**Sexual orientation**			
Lesbian / Gay / Homosexual	170 (40.96%)	11 (18.64%)	159 (44.66%)
Bisexual	73 (17.59%)	14 (23.73%)	59 (16.57%)
Straight/Heterosexual	132 (31.81%)	27 (45.76%)	105 (29.49%)
Queer	29 (6.99%)	4 (6.78%)	25 (7.02%)
Different identity	5 (1.2%)	2 (3.39%)	3 (0.84%)
Don’t know/Prefer not to answer	6 (1.45%)	1 (1.69%)	5 (1.4%)
**Country of origin**			
Australia	195 (46.99%)	42 (71.19%)	153 (42.98%)
Other country	217 (52.29%)	15 (25.42%)	202 (56.74%)
Not sure/Prefer not to answer	3 (0.72%)	2 (3.39%)	1 (0.28%)
**Highest level of education**			
Postgraduate level	119 (28.67%)	16 (27.12%)	103 (28.93%)
Bachelor level	163 (39.28%)	26 (44.07%)	137 (38.48%)
Diploma level	54 (13.01%)	3 (5.08%)	51 (14.33%)
Certificate level	36 (8.67%)	9 (15.25%)	27 (7.58%)
High School	42 (10.12%)	5 (8.47%)	37 (10.39%)
Others	1 (0.24%)	0 (0%)	1 (0.28%)
**Employment status**			
Student	61 (14.7%)	12 (20.34%)	49 (13.76%)
Full-time employment or self-employed	197 (47.47%)	19 (32.2%)	178 (50%)
Part-time /casual employment	108 (26.02%)	20 (33.9%)	88 (24.72%)
Retired	6 (1.45%)	0 (0%)	6 (1.69%)
Unemployed or not working	33 (7.95%)	6 (10.17%)	27 (7.58%)
Unable to work	3 (0.72%)	1 (1.69%)	2 (0.56%)
Other:	7 (1.69%)	1 (1.69%)	6 (1.69%)
**Previously tested for HIV/STIs**			
Yes	378 (91.08%)	44 (74.58%)	334 (93.82%)
No	34 (8.19%)	14 (23.73%)	20 (5.62%)
Not sure/Prefer not to answer	3 (0.72%)	1 (1.69%)	2 (0.56%)
**Comfort level using mobile apps and websites**			
Very comfortable	327 (78.80%)	49 (83.05%)	278 (78.09%)
Somewhat comfortable	61 (14.70%)	10 (16.95%)	51 (14.33%)
Neutral	19 (4.58%)	0 (0%)	19 (5.34%)
Somewhat uncomfortable	4 (0.96%)	0 (0%)	4 (1.12%)
Very uncomfortable	3 (0.72%)	0 (0%)	3 (0.84%)
Not sure/Prefer not to answer	1 (0.24%)	0 (0%)	1 (0.28%)
**Ever use an online risk assessment tool for STIs**			
Yes	58 (13.98%)	49 (13.76%)	9 (15.25%)
No	340 (81.93%)	292 (82.02%)	48 (81.36%)
Not sure/Prefer not to answer	17 (4.1%)	15 (4.21%)	2 (3.39%)

%: percentage

*IQR* interquartile range, *SD* standard deviation

^a^Online Recruitment: This includes recruitment from social media, including Facebook and X, the clinic's official website, and affiliated RACGP clinics

^b^Another gender not specified in the survey. Participants could also provide their gender in free text

### Preferences influencing the uptake of *MySTIRisk (Table S3)*

**Fig. 1:**
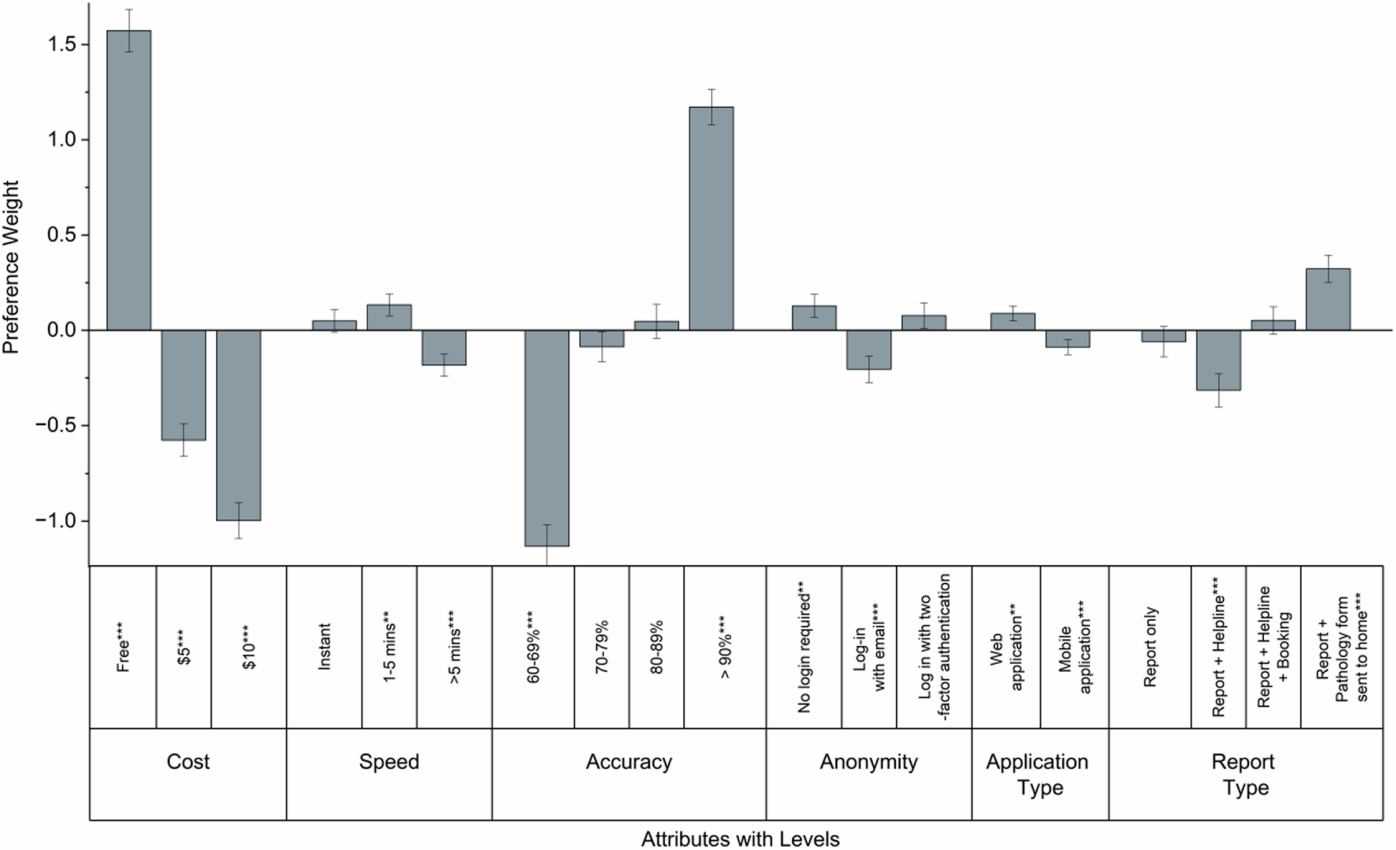
Utility Scores Distribution by Random Parameters Logit (RPL) model

**Fig. 2:**
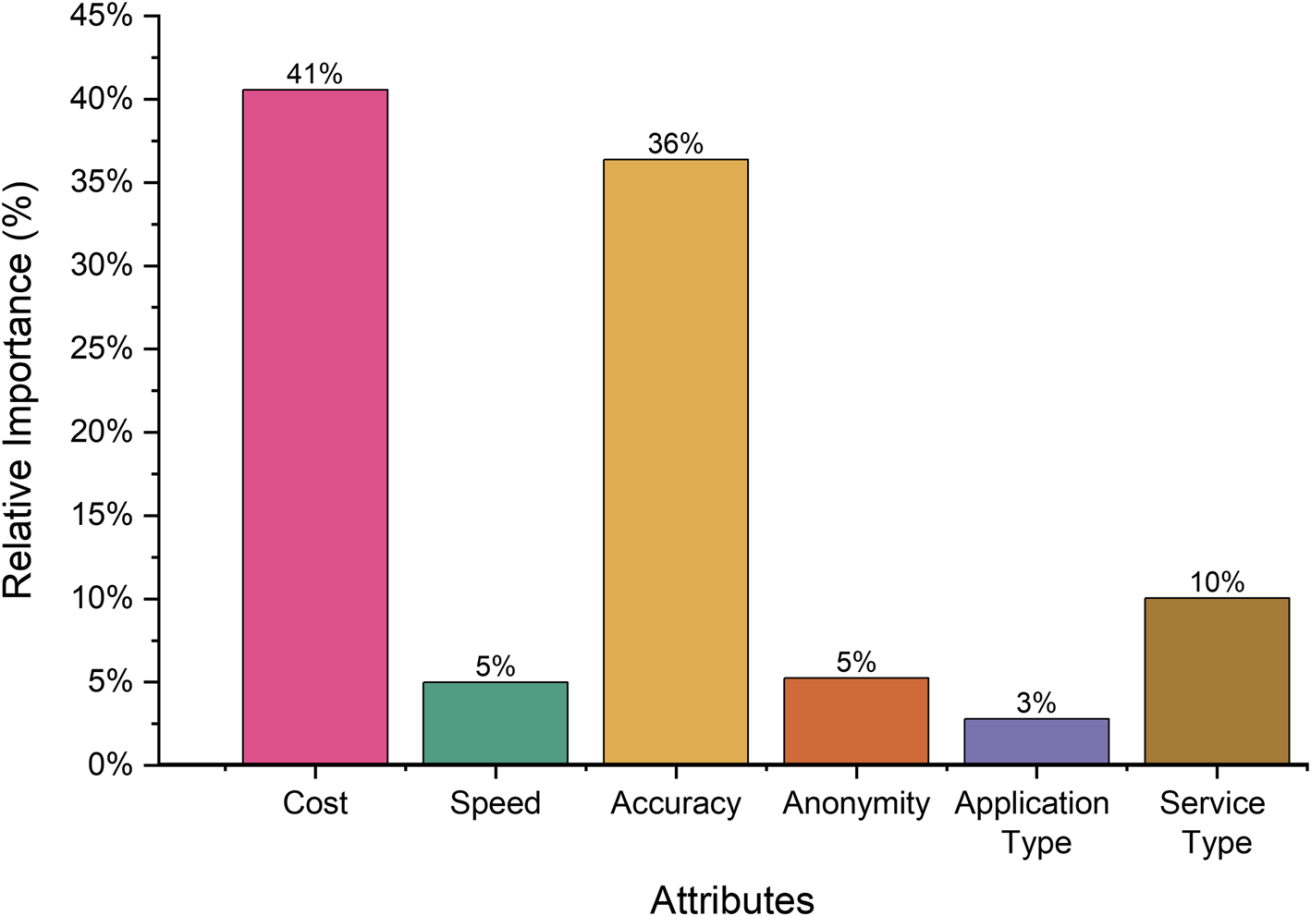
Relative Importance by Random Parameters Logit (RPL) model

The most influential attribute for the use of *MySTIRisk* was the cost (40.6%), followed by the accuracy of the tool (36.4%), the availability of additional services (10.1%), the level of anonymity (5.1%), the speed of receiving results (5.0%), and whether the tool was a web or mobile application (2.8%) (see Figs. 1 and 2). The most preferred tool was one that was free, with an accuracy above 90%, providing a report along with a pathology form sent to the user's home for HIV/STI testing, delivering results within 1–5 min, requiring no log-in, and being a web application without the need to download it. The least preferred tool was one costing AU$10, with lower accuracy (60–69%), offering only a report with a helpline option, taking more than 5 min to provide results, requiring a log-in with an email, and being a mobile application that requires downloading and installation (see Table 2 and Figure S1).

**Table 2:** Simulation scenarios and predicted uptake

Attributes	Current scenario (Status Quo)	Best scenario (with most preferred attribute levels)	Worst scenario (with least preferred attribute levels)
Predicted Uptake	91.9%	97.7%	22.3%
Cost	Free	Free	$10
Speed	Instant	1–5 min	More than 5 min
Accuracy	70–79%	> 90%	60–69%
Anonymity	No login required	No login required	Log-in with email
Application type	Web application	Web application	Mobile application
Additional services	Report only	Report + Pathology form sent to your home for you to do HIV/STI testing	Report + Helpline

### Preference heterogeneity revealed by latent class analysis

**Fig. 3:**
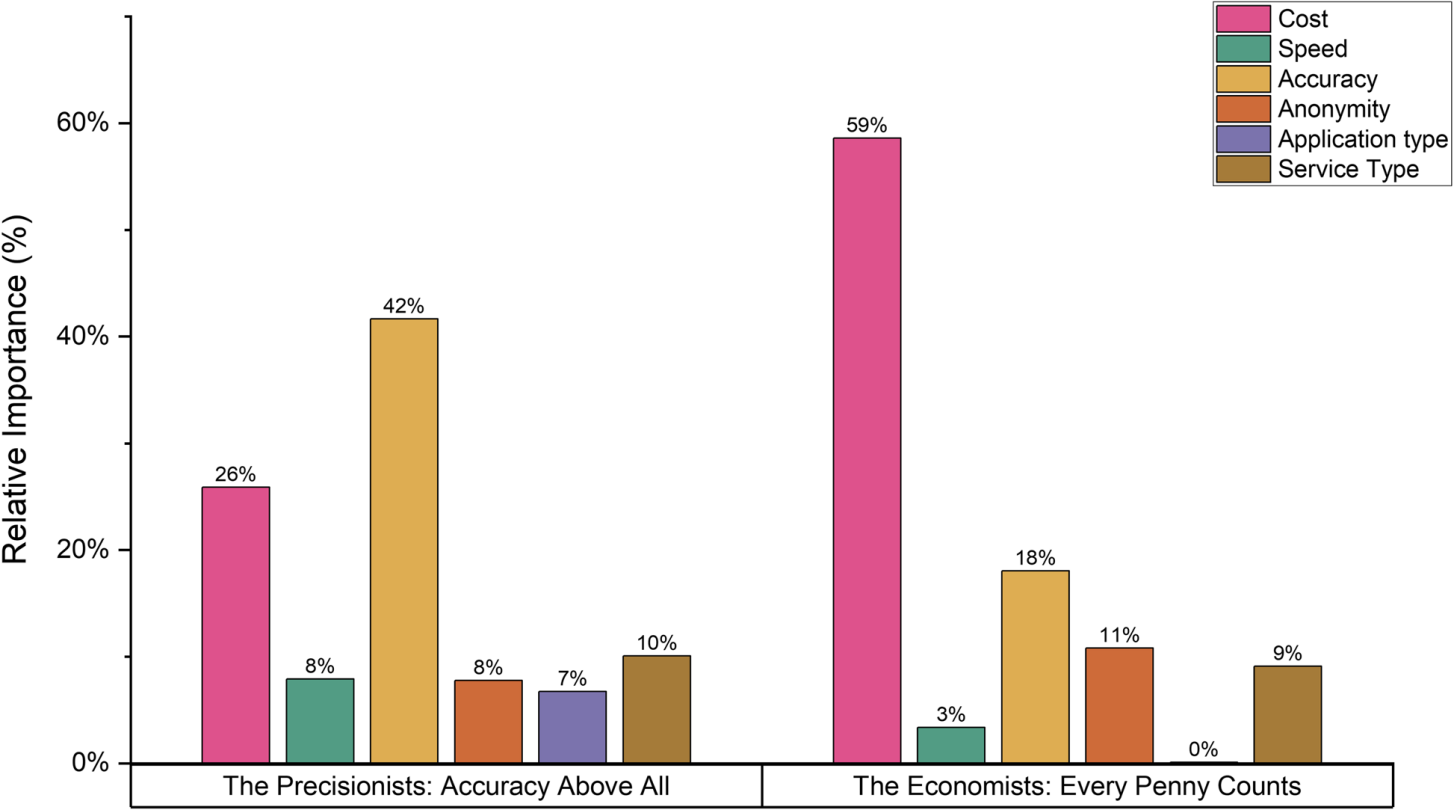
Relative Importance between Two Groups by the Latent Class Analysis

Table 3 shows the results of the latent class analysis (LCA). The relative importance of each attribute according to classes is shown in Fig. 3. The LCA findings indicate that the larger class (“The Precisionists", 66.0% of respondents) were mainly influenced by accuracy and were more likely to be participants who had recently arrived in Australia in the last 5 years. Despite their focus on accuracy, "The Precisionists" still preferred a free tool (*p* < 0.001), 1–5-min result times (*p* < 0.05), a web application (*p* < 0.01), and the inclusion of a pathology request form (*p* < 0.05). The second class (“The Economists", 34.0% of respondents) were mainly influenced by the free tool and were more likely to consist of individuals with higher education status (holding a bachelor's degree or above) and clients of MSHC. They preferred a tool with no log-in requirement (*p* < 0.01) and one that provides a pathology request form (*p* < 0.10). "The Economists" were less concerned about the accuracy of the tool compared to "The Precisionists".

**Table 3:** Latent class analysis of preferences for using an AI-powered risk assessment tool for HIV/STIs (*N* = 415)

	**"The Precisionists: Accuracy above all " (66.0%)**		**"The Economists: Every penny counts " (34.0%)**	
	**Coefficient**	**SE**	**Coefficient**	**SE**
Cost				
Free	0.70***	0.15	2.48***	0.18
$5	-0.11	0.15	-0.90***	0.19
$10	-0.59***	0.11	-1.58***	0.25
Speed				
Instant (less than 1 min)	-0.10	0.07	-0.11	0.17
1–5 min	0.23**	0.11	0.15	0.14
More than 5 min	-0.14	0.09	-0.05	0.14
Accuracy				
60–69%	-1.00***	0.09	-0.36*	0.20
70–79%	-0.06	0.16	-0.39*	0.20
80–89%	-0.03	0.19	-0.11	0.21
> 90%	1.09***	0.10	0.86***	0.17
Anonymity				
No login required	0.02	0.09	0.38***	0.14
Log-in with email	-0.20*	0.12	-0.37**	0.15
Log in with two-factor authentication	0.18	0.15	-0.02	0.15
Application Type				
Web application	0.17***	0.05	0.02	0.13
Mobile application	-0.17***	0.53	-0.02	0.13
Report Type				
Report only	-0.04	0.14	-0.23	0.24
Report + Helpline	-0.38**	0.15	-0.04	0.22
Report + Helpline + Booking system	0.10	0.15	-0.11	0.19
Report + Pathology form sent to your home for you to do HIV/STI testing	0.32**	0.13	0.38*	0.20
Theta in the class probability model				
Recent Arrival (< 5 years)	0.93***	0.28	Ref	
Highly Educated (Bachelor and above)	-0.62**	0.28	Ref	
MSHC clients	-0.88**	0.37	Ref	

AIC/*N* = 1.430, log-likelihood function = -1748.16

AIC indicates Akaike Information Criteria, *SE* standard error

^***^p value < 0.01

^**^p value < 0.05

^*^p value < 0.10

### Uptake of *MySTIRisk*

Simulation scenarios were used to estimate the potential uptake of *MySTIRisk* under various attribute combinations. In the best-case scenario, where the most preferred attribute levels were used (free, instant results, > 90% accuracy, no log-in required, web application, and a report with a pathology form sent to the user's home for HIV/STI testing), the predicted uptake was 97.7%. The current scenario, reflecting a more realistic set of attributes (free, 70–79% accuracy, no log-in required, web application, and a report only), yielded an uptake of 91.9%. However, in the worst-case scenario, with the least preferred attribute levels (AU$10, more than 5 min for results, 60–69% accuracy, logging in with email, mobile application, and a report with a helpline), the predicted uptake dropped to 22.3%. If we used the current scenario and only changed the cost per use of the *MySTIRisk* application, this would lead to an uptake of 67.8% (AU$5) and 58.7% (AU$10). If we used the current scenario and only changed the accuracy of the tool, this would lead to an uptake of 92.5% (80–89% accuracy) and 96.6% (> 90% accuracy).

## Discussion

Our discrete choice experiment (DCE) provides important insights into the preferences of potential users for an artificial intelligence (AI)-based HIV/STI risk assessment tool. To our knowledge, this is the first study to utilise a DCE to explore user preferences for an AI-powered risk assessment tool in the context of HIV/STI prevention. We found that cost and accuracy were the primary drivers influencing the uptake of such a tool, aligning with previous DCE studies on AI health applications [13, 15]. The strong preference for a free tool highlights the need to minimise financial barriers to access. High accuracy was also crucial, reflecting the importance of reliable risk predictions for health decision-making [7]. The latent class analysis further revealed distinct preference subgroups, with "The Precisionists" demanding high accuracy and "The Economists" prioritising low cost. Simulations predicted a high uptake (97.7%) for a tool designed with the most preferred attribute levels, contrasting sharply with the lower uptake (22.3%) for the least preferred design. These findings underscore the importance of accuracy and cost-free access for users to encourage the use of *MySTIRisk.* Our study represents the first step in potentially integrating *MySTIRisk* into public health strategies to increase testing for HIV/STIs and highlights the need for user-centric design in the development of health technology solutions [22].

Our study demonstrated that cost and accuracy were the primary determinants of potential users' willingness to use an AI-based HIV/STI risk assessment tool. The unequivocal demand for no-cost access underscores an essential barrier to widespread use, suggesting that even minimal fees could significantly deter potential users. Simulations using the current scenario with varying costs per use (AU$5 and AU$10) showed a substantial decrease in uptake to 67.8% and 58.7%, respectively, compared to the free version, highlighting the importance of cost-free access. This is consistent with prior research on HIV testing preferences, which has repeatedly identified cost as a key factor influencing testing decisions [23–25].

The strong preference for a free tool highlights the need for innovative funding models, potentially through government or healthcare organisations, to support the development and deployment of AI-based risk assessment tools to ensure equitable access [26, 27]. This is particularly important in Australia, where inequities in access to sexual health care disproportionately affect culturally and linguistically diverse (CALD) communities and those living in rural areas [28]. For instance, HIV rates are significantly higher among CALD communities, and access to sexual health services is limited for those outside urban areas [28]. Therefore, investing in free AI-based risk assessment tools like *MySTIRisk* could help address these inequities by increasing access to testing and early detection for marginalised populations.

The high importance placed on accuracy reflects the fundamental need for reliable risk assessments to inform sexual health choices and build user confidence in AI-powered interventions. It highlights the need to refine and update prediction models with new clinical data to ensure sustained accuracy and relevance. This involves incorporating user feedback, integrating comprehensive health data, and regularly assessing and updating the model to maintain user trust and ensure relevance in diverse health landscapes. This aligns with efforts to enhance personalised medicine approaches within public health, where AI can play a pivotal role in tailoring interventions to individual risk profiles [29]. Consequently, the preference for accuracy highlights a fundamental user demand and the need for robust mechanisms for ongoing model evaluation and enhancement [30]. This could involve regularly assessing the model's performance using clinically validated data, comparing predictions with actual outcomes, and updating the model with new data to improve its accuracy and generalisability. This iterative process is essential to maintain user trust and ensure the tool's relevance in diverse and changing health landscapes [31], ultimately contributing to the broader goal of reducing HIV/STI prevalence through targeted prevention strategies.

Beyond cost and accuracy, our study demonstrated preferences for additional features that, while not highly prioritised by users, enhance the user experience and could influence *MySTIRisk* uptake*.* Notably, users preferred the inclusion of pathology request forms, which facilitate testing and care. In Australia, individuals can obtain these forms from healthcare providers, and testing costs are typically covered by Medicare or private insurance. This preference aligns with the concept of integrated care, emphasising seamless transitions between risk assessment, testing, and treatment [32]. By streamlining the process, *MySTIRisk* can improve the user experience and potentially enhance public health outcomes by promoting timely testing and linkage to care.

Our findings highlight several avenues for future research to further optimise the development and implementation of AI-based HIV/STI risk assessment tools like *MySTIRisk*. First, given the importance of cost and accuracy in driving user preferences, future studies should focus on identifying the most cost-effective strategies for delivering highly accurate risk assessments. This may involve comparing different AI methodologies, data sources, and implementation models to determine the optimal balance between cost and performance. Second, the heterogeneity in user preferences identified through the latent class analysis suggests the need for further research on tailoring *MySTIRisk* to specific subgroups. Future studies could explore the feasibility and impact of offering customisable features or targeted versions of the tool based on user characteristics and preferences. For example, multilevel latent class analysis could be employed to identify distinct preference profiles within key sub-populations, such as gay, bisexual, and other men who have sex with men (GBMSM) and women of reproductive age. Finally, longitudinal research is needed to assess the real-world uptake, user satisfaction, and public health impact of *MySTIRisk* following its wide-scale promotion.

Our study has important implications for policymakers and healthcare organisations seeking to leverage AI-based tools to enhance HIV/STI prevention efforts. The strong preference for a free tool underscores the need for policies and funding mechanisms that support the development, deployment and continued maintenance of accessible, cost-free risk assessment resources. Governments and public health agencies should invest in tools like *MySTIRisk*, recognising their potential to expand testing access and promote early detection. Furthermore, the importance of accuracy highlights the need for regulatory frameworks and quality assurance processes to ensure that AI-based health tools meet rigorous standards of performance and are continuously monitored and improved based on real-world data. Furthermore, the participants' preference for features that facilitate practical next steps in care emphasises the importance of integrating AI health tools with existing healthcare services. Policies should encourage the development of AI tools that not only assess risks but also seamlessly connect users with testing, treatment, and care services. This integrated approach can significantly enhance public health outcomes by ensuring that individuals are not only informed of their risk but also guided towards timely and appropriate care.

Our study has several limitations that should be considered when interpreting the results. First, our findings may not accurately predict real-world behaviour, and an inherent gap between stated preferences and actual behaviour may exist, although previous studies suggest a reasonably close correlation between stated preferences in DCEs and real-world choices [33]. Second, while AI plays a crucial role in our risk assessment tool, it was not explicitly emphasised to users during the survey, as our study focused on tool features rather than AI perceptions. This limitation may have influenced respondents' preferences regarding the tool's features. Third, our recruitment efforts, though varied, resulted in a disproportionately large sample from the sexual health clinic. This approach, coupled with a notably low response rate, suggests that our findings represent the views of those more inclined towards participating in health research or those who are more health conscious. Moreover, due to the anonymous survey design, we could not directly compare the characteristics of responders to non-responders to evaluate non-response bias. While our latent class analysis explored the potential impact of these differences on user preferences, the small sample size of the online recruitment group limits the conclusiveness of these comparisons. Consequently, the generalizability of our results to the broader population will need further verification. To address this limitation, we are conducting external validation studies with participants from diverse populations outside MSHC. Additionally, we plan to implement *MySTIRisk* in community settings to assess its broader applicability. These studies aim to provide insights into the tool's performance and relevance across varied demographic groups. Fourth, while our LCA offered insights into preference heterogeneity, limitations in sampling (as acknowledged above) and not employing scale-adjusted models may have limited our ability to identify other distinct user subgroups. Lastly, the potential for social desirability bias, particularly among sexual health clinic attendees, cannot be overlooked. Participants might have reported preferences they perceived as more socially acceptable rather than their genuine choices. However, the survey's design was completely anonymous and completed in participants' own time, likely mitigating this bias.

Our study is the first to conduct a discrete choice experiment (DCE) specifically for an AI-powered risk assessment tool in the context of HIV/STI prevention. Despite these limitations, our study has several notable strengths. First, by employing a discrete choice experiment (DCE) methodology, we have provided novel insights into the preferences of potential users for an AI-based HIV/STI risk assessment tool, *MySTIRisk*. This approach goes beyond traditional survey methods by presenting participants with realistic scenarios that require trade-offs, providing richer data on user priorities. Second, by incorporating Latent Class Analysis (LCA), we were able to identify distinct user subgroups with varying preferences for cost, accuracy, and other attributes. This nuanced understanding of user heterogeneity allows for the development of more targeted strategies to promote *MySTIRisk* use among different user groups. Finally, the findings from this study provide valuable insights into user-centric design principles. By understanding user priorities and preferences, developers and public health agencies can develop AI-powered tools that are not only technologically advanced but also aligned with user expectations and needs.

The findings from our study have enhanced our understanding of user preferences for an AI-based HIV/STI risk assessment tool, emphasising the significance of cost and accuracy. This knowledge will inform future development of the tool, advocacy in policymaking around digital sexual health applications, further research on the tool's efficacy, and implementation in public health strategies.

## Supplementary Information

Supplementary Material 1.

Supplementary Material 2.

## Data Availability

The data that support the findings of this study are not openly available due to reasons of sensitivity and are available from the corresponding author, Professor Jason Ong, at Jason.ong@monash.edu, upon reasonable request. Data are in controlled access data storage at the Melbourne Sexual Health Centre.
