# Supplementary material for: Preferences for attributes of an artificial intelligence-based risk assessment tool for HIV and sexually transmitted infections: a discrete choice experiment
Source: BMC Public Health. 2024 Nov 21;24:3236. doi: 10.1186/s12889-024-20688-2 (PMC11580649; doi:10.1186/s12889-024-20688-2)
Supplement: Supplementary file 1 — Supplementary Material 1. [file 12889_2024_20688_MOESM1_ESM.docx]

Table S1- Attributes and levels of the discrete choice experiment

| **Attributes** | **Levels** |
| --- | --- |
| **Cost** | Free  $5  $10 |
| **Speed** | Instant (less than 1 min)  1-5 mins  More than 5 mins |
| **Accuracy** | 60-69%  70-79%  80-89%  > 90% |
| **Anonymity** | No login required  Log-in with email  Log in with two-factor authentication |
| **Application type** | Web application (no need to download application)  Mobile application (download the application) |
| **Additional services** | Report only.  Report + Helpline.  Report + Helpline + Booking system.  Report + Pathology form sent to home for you to do HIV/STI testing (explanation) |

Table S2: Example of a DCE choice set. Of the options presented below, which one would you choose? Please choose Option 1, Option 2 or None.


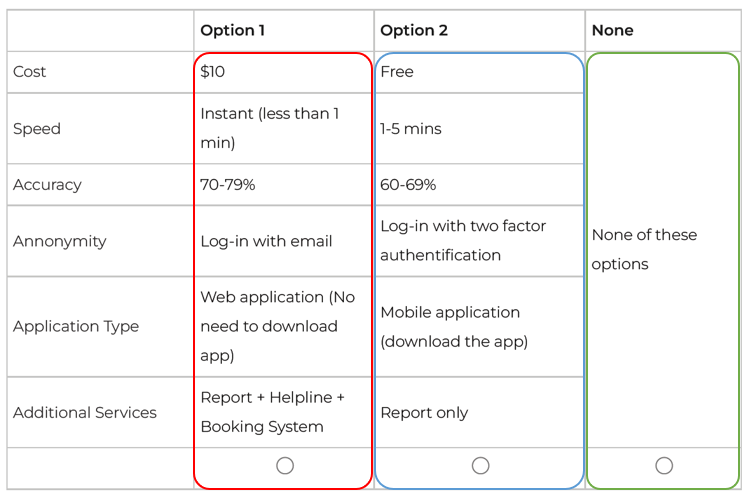


Table S3. Random parameters logit model of preferences for using an AI-powered risk assessment tool for HIV/STIs (N = 415).

| Attributes | Level | Coefficient (SE) | Standard deviation (SE) |
| --- | --- | --- | --- |
| Cost | Free | 1.55 (0.11) *** | 1.21 (0.14) *** |
|  | $5 | -0.58 (0.08) *** | 0.75 (0.11) *** |
|  | $10 | -1.00 (0.09) *** | 0.95 (0.1) *** |
| Speed | Instant (less than 1 min) | 0.05 (0.06) | 0.23 (0.32) |
|  | 1-5 mins | 0.13 (0.06) ** | 0.09 (0.22) |
|  | More than 5 mins | -0.18 (0.06) *** | 0.07 (0.25) |
| Accuracy | 60-69% | -1.13 (0.11) *** | 0.79 (0.35) ** |
|  | 70-79% | -0.09 (0.08) | 0.79 (0.19) |
|  | 80-89% | 0.05 (0.09) | 0.01 (0.25) |
|  | > 90% | 1.17 (0.09) *** | 0.02 (0.12) *** |
| Anonymity | No login required | 0.13 (0.06) ** | 0.02 (0.20) |
|  | Log-in with email | -0.20 (0.07) *** | 0.01 (0.11) |
|  | Log in with two-factor authentication | 0.08 (0.07) | 0.02 (0.16) |
| Application Type | Web application (no need to download application) | 0.09 (0.04) ** | 0.15 (0.12) |
|  | Mobile application (download the application) | -0.09 (0.04) *** | 0.15 (0.12) |
| Report Type | Report only | -0.06 (0.08) | 0.14 (0.33) |
|  | Report + Helpline | -0.31 (0.09) *** | 0.01 (0.13) |
|  | Report + Helpline + Booking system | 0.05 (0.07) | 0.03 (0.16) |
|  | Report + Pathology form sent to your home for you to do HIV/STI testing | 0.32 (0.07) *** | 0.14 (0.26) |
| Opt-out^#^ |  | -0.61 (0.71) *** |  |

AIC/N=1.665, log likelihood function= -2045.96

AIC indicates Akaike Information Criteria; SE, standard error.

*** p value <0.01.

** p value <0.05.

* p value <0.10.

^#^ Opt-out refers to a scenario where participants did not want to choose either Option 1 or Option 2.

*





*

Figure 1. Uptake Percentage (Simulation)

Figure 1. Uptake Percentage (Simulation)

Figure S1. Uptake Percentage (Simulation)
