# Supplementary material for: Preferences for attributes of an artificial intelligence-based risk assessment tool for HIV and sexually transmitted infections: a discrete choice experiment
Source: BMC Public Health. 2024 Nov 21;24:3236. doi: 10.1186/s12889-024-20688-2 (PMC11580649; doi:10.1186/s12889-024-20688-2)
Supplement: Supplementary file 2 — Supplementary Material 2. [file 12889_2024_20688_MOESM2_ESM.pdf]

Introduction

We are developing a tool to help members of the public assess their risk of acquiring HIV and other sexually transmitted infections (STIs), named "**MySTIRisk**".

We would like to invite you to participate in this short survey about your opinions of the features of the website MySTIRisk. This survey will take 10–15 minutes to complete.

You do not have to participate in this survey if you don't want to. This survey will not collect any information that can identify you. Your responses to this survey will remain confidential to the MSHC research team.

This study is approved by the Alfred Hospital Ethics Committee (Project number - 637/23). Please read the participant information sheet by clicking HERE.

If you agree to participate, please click on the "Agree" button below.
If you don't agree to participate, please click on the "Disagree" button below.

- ○ Agree
- ○ Disagree

Eligibility Questions

How old are you? (Write the number of years)

[ ]

Have you been sexually active with anyone within the **past twelve months**?

- ○ Yes
- ○ No
- ○ Unsure/Prefer not to answer

About MySTIRisk

The Melbourne Sexual Health Centre (MSHC) has developed a tool called **MySTIRisk** to **assess your risk** of HIV and other sexually transmitted infections (STIs) like syphilis, gonorrhoea and chlamydia. The tool uses computer programs that are referred to as 'artificial intelligence'. You can see example screenshots of the tool below.

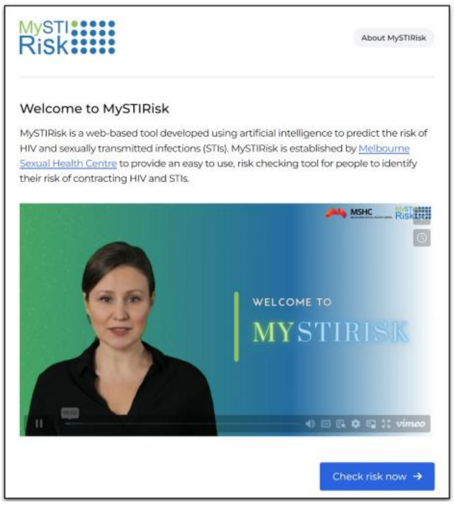

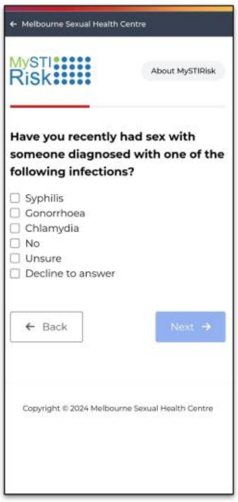

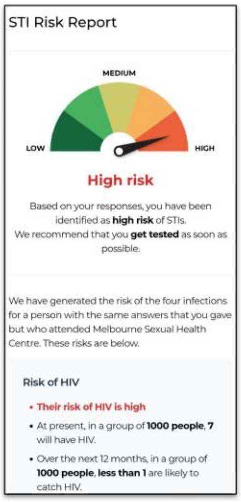

About this Survey

We would like to know **YOUR** preferences for the various features of the risk prediction tool "**MySTIRisk**". In the following section, you will be presented with various sets of options, each comprising different features of the tool.

Make sure you select **one complete set** of features—or "**package**"—that you prefer over the others in each question.

Please consider all the features of each option package carefully before making your selection. You should not pick and choose individual features from different options and combine them into a custom package; choose one complete package as it is presented. Your choices are invaluable to us and will significantly influence the design and functionality of MySTIRisk.

Of the options presented below, which would you choose? Please choose **Option 1**, **Option 2** or **None (See Example below).**

	Option 1	Option 2	None
Cost	$10	Free	None of these options
Speed	Instant (less than 1 min)	1-5 mins	
Accuracy	70-79%	60-69%	
Annonymity	Log-in with email	Log-in with two factor authentification	
Application Type	Web application (No need to download app)	Mobile application (download the app)	
Additional Services	Report + Helpline + Booking System	Report only	
	○	○	○

Imagine that you are worried you might have a sexually transmitted infection. You want to use an online risk prediction tool. Please let us know what version you prefer (**Option 1** or **Option 2**), or if you do not like either, please

pick **None** (i.e., you choose not to use this tool).

Conjoint Block (Auto-generated by Qualtrics)

(1/6) Choose your preferred option below:
(2/6) Choose your preferred option below:
(3/6) Choose your preferred option below:
(4/6) Choose your preferred option below:
(5/6) Choose your preferred option below:
(6/6) Choose your preferred option below:

How **easy** was it to **understand** the choices you had to make?

- ◯ Very Easy
- ◯ Easy
- ◯ Not easy or
- ◯ hard Hard
- ◯ Very hard

How **easy** was it to **answer** the choices you had to make?

- ◯ Very Easy
- ◯ Easy
- ◯ Not easy or
- ◯ hard Hard
- ◯ Very hard

Were there any characteristics you **mainly** paid attention to? [Tick all that apply]

- ☐ Cost
- ☐ Speed
- ☐ Accuracy
- ☐ A nonymity
- ☐ Application Type
- ☐ Additional Services
- ☐ I considered all characteristics before I made my choice

Are there any features that would be deal-breakers for you, making you **not** want to use the tool? [Tick all that apply]

- ☐ Cost per use ($5 per use)
- ☐ Slow tool (More than 5 mins to provide results)
- ☐ Lower accuracy
- ☐ Required login/identification
- ☐ Need to download the mobile application
- ☐ No additional support services
- ☐ None

Demographic Questions

What sex were you assigned at birth?

- ○ Male
- ○ Female
- ○ Intersex
- ○ Don’t know/Prefer not to answer

Which of the following best describes your current gender identity?

- ○ Male
- ○ Female
- ○ Non-binary/gender-fluid
- ○ Different identity, please specify: [ ]
- ○ Don’t know/Prefer not to answer

Do you consider yourself to be:

- ○ Lesbian / Gay / Homosexual
- ○ Bisexual Straight/Heterosexual
- ○ Queer
- ○ Different identity, please specify: [ ]
- ○
- ○ Don’t know/Prefer not to answer

Do you have sex with: [Tick all that apply]

- ☐ Men
- ☐ Women
- ☐ Non-binary
- ☐ Different identity, please specify: [ ]
- ☐ Don’t know/Prefer not to answer

Where were you born?

- ○ Australia
- ○ Other country
- ○ Not sure/Prefer not to answer

How long have you been in Australia?

- ◯ Less than 1 year
- ◯ Less than 5 years
- ◯ 5 years or longer
- ◯ Not sure/Prefer not to answer

What is the highest level of education you have completed?

- ◯ Postgraduate level
- ◯ Bachelor level
- ◯ Diploma level
- ◯ Certificate level
- ◯ High school
- ◯ Primary school
- ◯ [ ] Other (Please specify):
- ◯ Not sure/Prefer not to answer

What is your current employment status? (Tick all that apply)

- ◯ Student
- ◯ Full-time employment or self-employed
- ◯ Part-time /casual employment
- ◯ Retired
- ◯ Unemployed or not working
- ◯ Unable to work
- ◯ [ ] Other (Please specify):
- ◯ Not sure/Prefer not to answer

Have you ever been tested for sexually transmissible infections or HIV?

- ◯ Yes
- ◯ No
- ◯ Not sure/Prefer not to answer

How comfortable are you using mobile apps and websites?

- ◯ Very comfortable
- ◯ Somewhat comfortable
- ◯ Neutral
- ◯ Somewhat uncomfortable
- ◯ Very uncomfortable
- ◯ Not sure/Prefer not to answer

Have you used an online STI risk assessment tool before?

- ○ Yes
- ○ No
- ○ Not sure/Prefer not to answer

How satisfied were you with the previous risk assessment tool(s) you used?

- ○ Very dissatisfied
- ○ Dissatisfied
- ○ Neutral
- ○ Satisfied
- ○ Very satisfied
- ○ Not sure/Prefer not to answer

Voucher

Would you like to enter the prize draw to win a $50 gift voucher?

If "yes", a link where you will be prompted to enter your details will appear at the end of the survey.

- ○ Yes
- ○ No

Powered by Qualtrics
